# The Repertoire of Tissue Inhibitors of Metalloproteases: Evolution, Regulation of Extracellular Matrix Proteolysis, Engineering and Therapeutic Challenges

**DOI:** 10.3390/life12081145

**Published:** 2022-07-28

**Authors:** Salvatore Costa, Maria Antonietta Ragusa, Gabriele Lo Buglio, Simone Dario Scilabra, Aldo Nicosia

**Affiliations:** 1 Department of “Scienze e Tecnologie Biologiche, Chimiche e Farmaceutiche” (STEBICEF), University of Palermo, 90128 Palermo, Italy; salvatore.costa@unipa.it (S.C.); maria.ragusa@unipa.it (M.A.R.); gabriele.lobuglio@community.unipa.it (G.L.B.); 2 Proteomics Group of Fondazione Ri.MED, Research Department IRCCS ISMETT, Istituto Mediterraneo per i Trapianti e Terapie ad Alta Specializzazione, Via E. Tricomi 5, 90127 Palermo, Italy; sdscilabra@fondazionerimed.com; 3 Institute for Biomedical Research and Innovation—National Research Council (IRIB-CNR), 90146 Palermo, Italy

**Keywords:** TIMPs, ECM, inflammation, cancer, protein evolution, protein engineering, TIMP diversity

## Abstract

Tissue inhibitors of metalloproteases (TIMPs) belong to a fascinating protein family expressed in all Metazoa. They act as regulators of the turnover of the extracellular matrix, and they are consistently involved in essential processes. Herein, we recapitulate the main activities of mammalian TIMPs (TIMP1–4) in the control of extracellular-matrix degradation and pathologies associated with aberrant proteostasis. We delineate the activity of TIMPs in the control of extracellular matrix (ECM) homeostasis and discuss the diversity of TIMPs across metazoans taking into account the emergence of the components of the ECM during evolution. Thus, the TIMP repertoire herein analysed includes the homologues from cnidarians, which are coeval with the origins of ECM components; protostomes (molluscs, arthropods and nematodes); and deuterostomes (echinoderms and vertebrates). Several questions, including the maintenance of the structure despite low sequence similarity and the strategies for TIMP engineering, shed light on the possibility to use recombinant TIMPs integrating unique features and binding selectivity for therapeutic applications in the treatment of inflammatory pathologies.

## 1. Introduction

Tissue inhibitors of metalloproteases (TIMPs) are a family of proteins that modulate the turnover of extracellular matrix (ECM) components by inhibiting the activity of matrix metalloproteases (MMPs) and the related “disintegrin and metalloproteases with thrombospondin motifs” (ADAMTSs) [1]. In addition, TIMPs regulate the release of cell-membrane proteins, a process known as ectodomain shedding, by inhibiting “disintegrin and metalloproteases” (ADAMs) [2]. The aberrant activity of such metalloproteases has been associated with several pathological conditions; therefore, their tight regulation by TIMPs is critical to maintaining proteostasis and thus physiological ECM turnover and cell signalling [3,4,5]. Four TIMPs are expressed in mammals. All of them display a wedge shape that perfectly complements the active-site cleft of metalloproteases and comprises two separate domains, the *N*- and *C*-terminal domains [2]. While the N-terminal domain is necessary to insert the metalloprotease active-site cleft and inhibit the enzyme, the functions of the C-terminal domain are still largely unknown.

All mammalian TIMPs possess two distinct domains, the N-terminal domain, of about 125 amino-acid residues, and the C-terminal domain, with about 65 residues [6]. The N-domain encompasses a five-stranded β-barrel with the Greek-key topology corresponding to the Oligonucleotide/Oligosaccharide-Binding Fold (OB-fold), while the C-terminal domain is composed of two parallel and two antiparallel strands followed by a helix. A helical region is located at the interface of the domains. Three disulfide bonds stabilize the structure; the cysteines in the Cys1-X-Cys3 sequence are bonded with two conserved cysteines, namely, Cys70 and Cys100, respectively (numbering according to the TIMP-1 sequence), while the third Cys, residue 13, is disulfide-bonded with Cys123 [6].

Moreover, TIMPs harbour a Cysteine 1–Xaa residue–Cysteine 3 motif at the N-terminal region of the mature protein that is crucial for coordinating the proteolytic Zn^2+^ ion and blocking the catalytic activity of the enzyme. Indeed, the mechanism of inhibition mediated by TIMPs exploits their conserved Cys1, which coordinates the Zn^2+^ ion in the active-site cleft of the enzyme, thus displacing the H_2_O molecule required for hydrolysis [7]. Nevertheless, despite their conserved wedge-like structure and mechanism of action, each of the four mammalian TIMPs harbours structural determinants that underlay a different metalloprotease inhibitory profile and unique biological functions [1,2].

Under physiological conditions, ECM-component proteolysis and turnover are regulated by the balance between MMPs and TIMPs, and the alteration in the ratio expressed, MMPs/TIMPs, is associated with pathological conditions associated with increased ECM deposition (fibrosis); boosted ECM degradation, such as that which occurs in several inflammatory diseases including arthritis; cancer establishment and progression; and cardiovascular disorders [8,9].

TIMPs are expressed in all metazoans, and their evolution appears to be dynamically represented by a set of complex processes of amino-acid-residue rearrangement. However, TIMPs have maintained a common 3D wedge-like structure throughout evolution, from cnidarians to humans [10]. Such structural conservation is coherent with the key role of TIMPs in essential physiological activities. 

During evolution, TIMPs have acquired different functions that are driven by different structural determinants. In this review, we summarize the evolution of TIMPs over 600 Myr, spanning from cnidarians to vertebrates, which unveils the relationship between the evolutionary rate of a region and the structural constraints ensuring the conservation of a prototypic structure. 

Despite the fact that the four human TIMPs may act as broad-spectrum MMP inhibitors, functional specialization for metalloproteinase inhibition usually occurs, which results in the restriction of the inhibitory range. On the basis of the pattern of expressed TIMPs in defined pathological alterations, several attempts have been made in the evaluation of the therapeutic potential of TIMPs [11,12]. 

In this review, we summarize the sequence and structure evolution of TIMPs relating them to their functionality and human pathologies. Then, we discuss how studying the evolution of TIMPs can lead to the development of molecules that may find therapeutical applications on selected pathological alterations. Finally, we propose an experimental pipeline for the functional evaluation of the inhibitory activity of specific TIMP members towards mammalian MMPs, ADAMs and ADAMTs in evolutionarily distant organisms.

## 2. The Role of TIMPs in the Regulation of ECM-Component Turnover and Pathologies

TIMPs, as inhibitors of MMPs, ADAMs and ADAMTSs, act as a pivot in the control of ECM proteolysis. The first piece of evidence suggesting this function arises from the in vitro ability of TIMPs to inhibit several MMPs and in the accumulation of matrix in a pathophysiological context associated with TIMP overexpression, as it occurs in idiopathic pulmonary fibrosis [13,14,15,16]. As a general rule, the balance between MMPs and TIMPs is thought to be responsible for ECM homeostasis. Thus, a shift in the balance in favour of MMPs boosts ECM-component modifications and turnover; conversely, increased TIMP activity results in the protection of the ECM. 

Humans are known to express four TIMP isoforms, namely, TIMP-1, -2, -3 and -4, with molecular weights ranging from 20 to 23 kDa in mature forms. As reported above, all human TIMPs harbour two distinct domains, the N-terminal domain, consisting of about 125 amino-acid residues, which is known to possess the full inhibitory activity, and a smaller C-terminal domain, which has been reported to affect the affinity of TIMPs for MMPs and ADAMs [17,18]. Despite these structural features being common to all the isoforms, the four mammalian TIMPs display distinct inhibitory profiles. While TIMP-2 and TIMP-4 are able to inhibit most MMPs, TIMP-1 shows a reduced inhibition of membrane-type MMPs and possesses the ability to attenuate the degradation of the extracellular matrix in healthy tissue as well as under pathological conditions. Analyses of the Timp1-/- mice phenotype have shown the role of TIMP1 in the preservation of normal myocardial structure and function via the control of fibrillar-collagen content [16].

Multiple studies have reported the role of TIMP2 in the inhibition of ECM proteolysis in several tissues and pathological assets [19,20,21]. Among them, an excess of ECM deposition in the hand resulting in the fixed flexion of joints characterizes Dupuytren’s syndrome. In this pathological condition, an aberrant ratio of TIMP2 and MMP2 in favour of TIMP2, which causes the inhibition of ECM proteolysis, is observed [22,23]. Similar toTIMP1, TIMP2 has a role in the control of the deposition of the ECM in several pathologies associated with heart disease, especially through the inhibition of MMP14 [24,25]. However, TIMP2 is also known to possess a dual role; indeed, in association with MMP14, it is required for the activation of MMP2 [25]. 

TIMP-3, in addition to MMPs, inhibits several members of the ADAM and ADAMTS families. Thanks to its broader inhibitory profile, TIMP-3 has been proven beneficial in diseases such as rheumatoid arthritis [26], which is majorly driven by an excess activity of TIMP-3 target ADAM17, and osteoarthritis, which is characterized by cartilage breakdown due to ADAMTS-4 and -5 [27].

TIMPs possess several functions other than the direct regulation of ECM proteolysis. This is because MMPs do not merely act on ECM components but also on cytokines, chemokines and cell-surface proteins, thus inducing general changes such as changes in inflammation, innate system and ectodomain shedding [28,29].

In addition to controlling ECM turnover via MMP inhibition, several reports have provided evidence for TIMP-1’s tumour-promoting effects, which occur via cell signalling independent of MMP inhibitory activity [30,31,32,33]. Concurrently, TIMP-1 expression has been found to be related to therapy resistance and poor prognosis in several cancers [34,35,36]. The involvement of TIMP-2 in oncological transformation has also been well documented, as it can regulate signalling pathways via the direct interaction with the cell-surface receptors on normal and cancer cells as well as the altering of the STAT3 pathway [37,38,39]. The anti-angiogenic effects of TIMP-2 have also been reported; in particular, the inhibition of endothelial cell migration mediating α3β1 integrin has been shown [40]. Recent efforts have also shown that TIMP-2 may act by suppressing TNBC (triple-negative breast cancer) growth and regulating the epithelial–mesenchymal transition [41]. The downregulation of TIMP-3 expression has been observed in several tumours, thus suggesting that it plays a tumour-suppressive role [42,43,44,45], and accordingly, it has been demonstrated that TIMP-3 expression induces apoptosis in cancer cell lines and reduces the invasiveness of cancer cells in vitro [46,47]. Similar to TIMP2, TIMP-3 has been also described as an angiogenesis inhibitor [11]. Studies have also reported that TIMP-4 is expressed in some cancers; however, its role in cancer establishment and/or progression remains unclear [11]. Recently, it has been shown that in nude mice, cervical cancer cells that overexpress TIMP-4 form tumours faster than the controls, thus providing evidence for a TIMP-4 related regulation of stemness [48].

## 3. The Diversity of TIMP Homologues

Over the years, several efforts have been performed for identifying the members of the TIMP superfamily in model and non-model organisms. Large-scale genome and transcriptome dataset collections available on public databases represent useful resources for the mining of gene products, complementing biochemical or canonical molecular identification procedures. Based on the exploitation of advanced search tools implementing the motif scan of key amino-acid-residue distributions that are characteristic of a specific target, strategies for in silico identification are available. Taking advantage of the availability of the growing number of sequenced genomes and transcriptomes and implementing advanced tools for sequence similarity searches with in silico 3D protein modelling, Nicosia et al. [10] have carried out an evolutionary survey on different taxonomic divisions. The results are a picture showing the homologs of TIMPs that are widely spread in species belonging to either vertebrates or invertebrates and that the evolution of TIMPs is closely connected to and cannot be considered separately from the evolution of the extracellular matrix (ECM).

**Figure 1:**
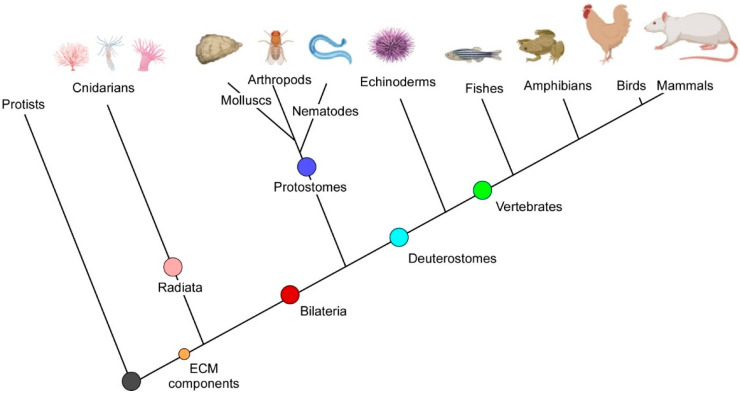
Eumetazoa phylogeny. The diagram represents the relationships among taxa; the branch lengths do not account for evolutionary distances among species. The main taxonomic divisions are indicated as well as the appearance of the ECM toolkit along with its evolution [10].

The evolution of multi-cellular organisms from single cells allowed more complex organisms (Metazoa) with cellular diversity and mechanisms of cell differentiation leading to specialized tissues to appear. The extracellular matrix acts as a pivot in such a process, contributing to spatial cell organisation and 3D arrangements of cells by binding adhesion receptors located on the membranes of the cell surface and congruently supporting the survival of cells in tissue organization [49]. ECM activity also relies on processing enzymes, including extracellular proteases, that modify the proteinaceous network, enabling all the specialised functions required in metazoans. Based on genomes and RNAseq data, it is possible to conclude that ECM-encoding genes originated before the appearance of radiated symmetry in the eumetazoan evolution (Figure 1). The Cnidaria phylum is the sister taxa of Bilateria, and some studies have demonstrated that it diverged from the lineage leading to bilaterians about 600 million years ago [50,51].

Existent cnidarians are simple aquatic organisms that show a basic diblastic organization consisting of two cell layers, namely, the ectoderm and the endoderm, separated by the mesoglea, which constitutes an ECM resembling the basal lamina containing type IV collagen [52,53].

Interestingly, MMP-dependent mechanisms of mesoglea remodelling have been described, at least in *Hydra* [54,55,56,57]. Despite diploblastic organization, histological and experimental evidence for mesodermal differentiation supports the hypothesis that these organisms are reduced Mesodermata representing an important step in the early evolution of the mesoderm and tissue organization [58]. Thus, cnidarians have been described as an evolutionary crossroads in developmental biology [59].

In this scenario, it is not strange that *Hydra* represents a model for studies on cell–ECM interactions that involve mechanisms of mesoglea remodelling via the digestion of ECM components related to MMPs [54,55,56,57], especially during morphogenesis, regeneration and transdifferentiation [60,61]. 

Only a few data are available on mechanisms leading to the control of mesoglea integrity via ECM-component turnover in cnidarians [62]. However, it should be noted that Leontovich et al. have demonstrated that the use of recombinant human TIMP-1 is able to inhibit *Hydra* MMPs, thus confirming the maintenance of a function even through evolutionary distant counterparts [54]. It is sufficiently reasonable that TIMPs regulate the remodelling of cnidarian ECM components. 

Although cnidarians are known to have diverged from Bilateria earlier than protostomes and deuterostomes (Figure 1), *Hydra magnipapillata*, *Nematostella vectensis* and *Acropora millepora* express several TIMPs, either as single- and double-domain homologs [50,63]. Thus, it appears that an ancestral two-domain TIMP was already extant before the eumetazoan radiation >600 Ma. Usually, the 3D structures of N-domains present a closed b-barrel composed of five or six b-strands with Greek-key topology, while C-terminal domains are organized in two parallel and two antiparallel strands followed by an α-helix. A region organized in α-helices is located at the interface of these two domains [10].

Among protostomes, nematodes (*Caenorhabditis elegans*) also express multiple TIMPs as single-domain proteins corresponding to the N-terminal inhibitory domain, and molecular phylogenetic analyses indicate that TIMPs from *C. elegans* cluster with single-domain homologues from *Hydra*. Double-domain proteins have been identified in different branches of protostomes, including *Drosophila melanogaster* [64] and blood clam *Tegillarca granosa* [65]. 

As hypothesized by Brew et al. [2], because of the dispersed distribution of single- and double-domain TIMPs, the single-domain protein cannot represent a model for the ancestral protein, and the moment in which double-domain proteins appeared cannot be established. Furthermore, on the basis of TIMP domain organization and evolutionary relationships among species, it has been proposed that TIMP evolution is not a linear process.

It has been reported that, despite low sequence similarity, amino-acid substitutions are accepted during evolution in such a way that satisfies the restraints arising from structure and function. Thus, secondary structure elements (SSEs), namely, α-helices and β-strands, which organise the two domains of TIMPs, and concurrently, the 3D structures result maintained [10]. Occasionally, short stretches of amino-acid residues structured in unusual α-helices in the N-domain of proteins appear in some TIMPs (see Table 1). Several authors have reported that differences occur in the representativeness of SSEs among conserved and random regions in different sets of proteins [66,67]. In this case, as a general rule, helices are found to be underrepresented in conserved regions, while β-strands are more likely to be absent in variable regions [68]. Additionally, other structural constraints related to evolutionary features define the correlation between the maintenance of a function and the acceptance of sequence variability in a specific region. Among them, the Relative Solvent Accessibility (RSA) of a residue in a protein measures the extent of the burial or exposure of that residue in the 3D structure. In this light, it has been shown that for a specific residue, the exposure to the solvent is anticorrelated with conservation [69,70,71,72].

On this basis, Nicosia et al. [10] have computed the RSA for all the sequences reported in Table 1, inferring that the stretches of amino-acid residues that organise the unusual α-helices in the N-domain of TIMPs are more likely to possess an exposed propensity so as to localize on the surface of the proteins providing tolerance to amino-acid substitutions. Thus, it is argued that under these conditions, the observed sequence variability is allowed to accumulate without impairing the N-domain’s ability to fold as a wedge and maintain inhibitory activities.

Based on such evidence, it is supposed that the evolution of TIMP diversity and architectures could be ascribed to the existence of distinct gene lines coding for single- and double-domain proteins. In this line, events of gene duplication, domain loss and nucleotide substitutions may represent the different pathways driving the evolutionary forces diversifying the TIMP repertoire.

**Figure 2:**
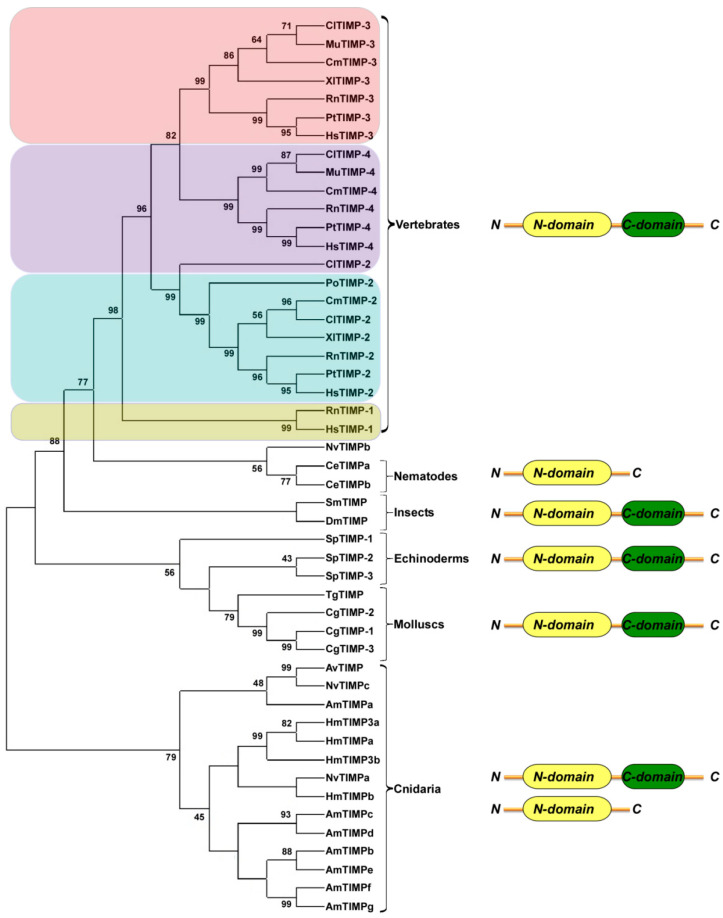
NJ phylogenetic tree based on the TIMPs from cnidarians to vertebrates. The tree was generated using MEGA X. All the sequences used were obtained from GenBank at National Centre for Biotechnology Information (NCBI). The p-distance model was used to construct the phylogenetic tree. Internal branches were assessed using 1000 bootstrap replications. Bootstrap values greater than 40% are indicated at the nodes. Gene nomenclature is according to [10]. The main taxonomic divisions are indicated. Single- and double-domain TIMPs are shown next to the corresponding groups Different colors represent TIMPs clustering according to isoforms in vertebrates.

Brew and Nagase [2] and Nicosia et al. [10] have reported that the evolution of TIMPs (Figure 2) in Cnidaria, protostomes and echinoderms seems to have ensued mainly according to taxa distinction, while homologs in Vertebrata branched off as polyphyletic groups, despite the fact that the considered TIMPs belong to different species. Thus, it is argued that the occurrence of gene duplication was followed by convergent evolution. Additionally, vertebrate TIMP-1 has been shown to most likely be the ancestral form; conversely, TIMP-4 appears as the most modern one.

## 4. TIMPs: Explore the Past to Understand the Present and Shape the Future

TIMPs are a group of endogenous inhibitors of metalloproteases whose major function is modulating the turnover of ECM components. TIMPs are expressed in all metazoans, and evidence suggests their role in modulating ancestral ECMs, in the cnidarian mesoglea and in the regulation of ectodomain shedding [73]. Although metazoan TIMPs show a conserved wedge-like shape across evolutionarily distant species, some structural features are unique for specific TIMPs. As discussed above, double-domain TIMPs have been identified in different branches of the phylogenetic tree, including invertebrates, such as *Drosophila melanogaster* [64] and blood clam *Tegillarca granosa* [65], and vertebrates, such as Japanese flounder *Paralichthys olivaceus* [74], fugu [75] and grass carp *Ctenopharyngodon idella* [76]. Interestingly, several functional single-domain TIMPs are expressed in older animal phyla, including cnidarians and nematodes. However, these phyla also express double-domain TIMPs, thus supporting the hypotheses that single- and double-domain TIMPs may (i) regulate different cohorts of MMPs, (ii) carry out different physiologic activities and (iii) be expressed at different moments. All these aspects could contribute to the arranging of the complex network of activities exploited by the members of this family.

Dissecting the structure and function of TIMPs from different metazoans may aid in identifying the structural determinants that drive specific TIMP functions in humans. 

Many pathological conditions in humans, including pulmonary fibrosis; cancer; rheumatoid arthritis; osteoarthritis; encephalomyelitis; heart, lung and kidney diseases; and diabetic nephropathy, are associated with the uncontrolled activities of MMPs, ADAMs and ADAMTSs linked to aberrant ECM breakdown or the excessive deposition of ECM components leading to fibrosis. Thus, over the years, the possible use of chemical MMP inhibitors with potential clinical applications has been hypothesised. However, MMP structures and active sites closely resemble each other; additionally, they usually exert overlapping roles in physiological processes. This could explain how synthetic MMP inhibitors have failed in early clinical trials [77]. 

An alternative approach to overcome problems arising from the use of broad-spectrum inhibitors in the treatment of metalloproteinase-related diseases is represented by the design of engineered TIMPs with restricted inhibitory specificities [78]. Thus, the possible use of TIMPs as therapeutic targets has been hypothesised. In this scenario, TIMPs are considered as potential therapeutics or targets to be used in the treatment of different pathologies.

As stated above, based on MMP structural and functional superposition, TIMPs per se are broad-spectrum inhibitors; however, X-ray crystallography and NMR studies have defined the three-dimensional structures of TIMPs and their complexes with MMPs. As a result, it has been established that they differ in the types of interactions via which TIMPs engage with the different MMPs analysed [79,80]. In addition, it has been defined that residue 2 of TIMP, Threonine (Thr), interacts with the S1’ pocket of MMPs, thus greatly influencing the affinity for the different MMPs [80]. This suggests that efforts for engineering their affinities toward a specific array of MMPs can be sustained by mutagenesis and recombinant-DNA technology.

An earlier study has shown that the conversion of Thr2 into Ala results in a restriction of TIMP-1 inhibitor activity, which is less effective towards MMP-1 if compared with MMP-2 and MMP-3 [81]. Additionally, TIMP1 mutants with substitutions at residues 4 and 68 have been combined with the previously studied Thr 2 mutations so as to generate mutants with improved selectivity for or binding affinity to specific MMPs. Mutations in residues 2, 4 and 68 produce an engineered TIMP-1 variant that effectively inhibits MMP-2 and MMP-3 but does not exert any action on MMP-1 [82], while for the conversion of TIMP-1 into an active inhibitor against MT1-MMP, the mutation of a single residue, namely, Thr98 to leucine, is required [83].

A specific MT1-MMP TIMP1 inhibitor, Valine (Val)4 Alanine (Ala)/Proline (Pro)6 Val/Thr98 Leucine (Leu), has been engineered by fusing TIMP-1 to a glycosyl-phosphatidyl inositol (GPI) anchor to target the plasma membrane, thus achieving a membrane-tethered high-affinity TIMP 1 variant that is more effective for MT1-MMP inhibition [84]. 

The mutation of Thr2 to Glycine that does not possess a lateral chain leads to a TIMP-1 variant able to act on MMP-9 rather than on MMP-2. Conversely, the conversion of Thr2 into Arginine (Arg) and the substitution of the TIMP-1 AB loop with the TIMP-2 AB loop result in a TIMP-1 variant with selectivity for MMP-2 [85]. 

TIMP-2 has also been mutagenised in order to select variants of human TIMP-2 that are selective inhibitors of human MMP-1. In this line, double mutant Serine (Ser)2 Aspartate (Asp)/Ser4 Ala results effective on MMP-1; however, an off-target basal inhibitory activity is retained on MMP-3, MMP-7 and MT1-MMP [86]. Interestingly, on the basis of structural and mutational analyses of the interactions involving TIMP-2 with the different MMPs analysed, it has been argued that TIMP-2 per se does not possess specific cues that drive the interaction with any specific MMP; therefore, multiple and combinatorial mutations affecting TIMP2 affinity may result in an interesting pattern of specificity [87]. The combinatorial engineering of N-TIMP2 variants has been carried out for the selection of variants that are able to selectively inhibit MMP9 and MMP14. TIMP-2 variant Ser4 Asp/Isoleucine (Ile)35 Leu/Asparagine (Asn)38 Ser/Ser68 Asp/Val71 Ser/Histydine (Hys)97 Ser/Thr99 Phenylalanine (Phe) possesses enhanced specificity for MT1-MMP rather than for MMP9, while TIMP-2 mutant Ser4 Proline (Pro)/Ile35 Pro/Asn38 Trp/Ser68 Asn/His97 Lysine (Lys)/Thr99 Lys works in the opposite manner [88]. Computational methods and yeast-surface-display techniques have been used to obtain highly specific inhibitors of MT1-MMP; in particular, TIMP-2 variant Ile35 Metionine (Met)/Asn38 Asp/Ser68 Asn/Val71 Gly/His97 Arg results in optimal interactions with MT1-MMP avoiding target binding with MMP-2 and MMP-10 [89]. 

The use of recombinant TIMP-3 has been shown to inhibit cartilage degradation both in vitro [86] and in vivo [87], confirming TIMP-3 chondroprotective activity under osteoarthritis conditions. TIMP-3 has been proven beneficial in diseases such as rheumatoid arthritis [23], which is majorly driven by an excess activity of TIMP-3 target ADAM17, and osteoarthritis, which is characterized by cartilage breakdown due to ADAMTS-4 and -5 [24]. Subsequently, the potential therapeutic treatment of recombinant TIMP-3 forms has been considered. 

Nevertheless, despite its wide inhibitory spectrum and thus its ability to inhibit additional metalloproteases mainly involved in arthritis, TIMP3 therapeutic use has limited application in clinics. In order to reduce the risk of mechanism-based side effects, TIMP-3 has been engineered to enhance its selectivity towards ADAM17, ADAMTS-4 and -5 [88]. In addition, different approaches have been used to decrease its turnover and potentiate its protective effects in tissue [89].

Although it has been shown that the amino-acid residues at the N-terminal domain of TIMPs located between Cys1 and Cys70 (according to human TIMP-1 residue numbering) account for ∼75% of the contacts with MMPs, the C-domain also affects MMP specificity, as shown by the expression of a recombinant TIMP-1 possessing the TIMP-2 C-terminal domain. Chimeric TIMP1 shows increased inhibitor effects against MT1-MMP and MMP-19 compared with wild-type TIMP-1 [17]. Additionally, considerations in the design of mutant TIMPs as selective metalloproteinase inhibitors take advantage of the definition of the residues located either at the *N*- or *C*-terminal domains of TIMP-1, which modulate the interaction with MMP-3 [18]. 

Other than providing important information about the evolution of these tissue-protective proteins, investigating TIMPs from evolutionarily distant species may lead to the identification of TIMPs with unusual dispositions of key residues, which in turn may affect affinity for and binding with human MMPs. Thus, the unique inhibitory properties that these TIMPs may possess could potentially be used in the therapy of diseases associated with aberrant ECM breakdown, including arthritis. 

The pipeline for the bioprospecting of candidate-TIMP identification encompasses several steps, as explained below.

i. Database analyses by motif and/or the homology screening of the specific distribution of key amino-acid residues or the selection of matching sequences on the basis of the occurrence of specific parameters as regional and structural constraints in the mapping of precise amino-acid residues, including the secondary structural elements and local solvent accessibility that are required for MMP inhibition and ECM interaction.
ii. The recovery of matching sequences, sequence optimisation via codon-bias removal and gene synthesis for optimal mammalian expression.
iii. Cloning into appropriate mammalian expression vectors and subsequent functional testing on different cell lines. This includes the analyses of the inhibitory profile of selected TIMPs towards members of human MMPs, ADAMs and ADAMTSs; collagen-degradation assays for those TIMPs with high selectivity for collagenases (MMP-1, MMP-8, MMP-13); aggrecan-degradation assays for those TIMPs with high selectivity for ADAMTSs; and APP- or TNF-shedding assays for TIMPs inhibiting ADAM10 or ADAM17, respectively.
iv. Finally, because the half-life of TIMP-3 in tissue is positively regulated by interactions with components of the ECM and negatively regulated by endocytosis and lysosomal degradation, which occur via interactions with scavenger receptor LDL receptor-related protein-1 (LRP-1) [90], the identification of selected TIMPs with reduced affinity for LRP1 would result in increased MMP inhibition.

## 5. Conclusions

Because of the involvement of TIMPs in several diseases and their roles in the control of ECM homeostasis via the inhibition of MMP-related proteolysis, data elucidating TIMP functions and biological activity are continuously produced, and as a result, more than 3900 published papers can be retrieved by interrogating PubMed with a 5-year window restriction. Among them, a considerable number of reports discuss or develop engineered TIMP variants in order to reduce the broad-spectrum-inhibition propensity and concurrently obtain a restricted inhibitory range for all TIMPs. Recent advances do not merely consider modifications and substitutions of amino-acid residues located at the N-domain, but they encompass the overall structure, because the *N*- and *C*-terminal domains cooperate in matrix-metalloproteinase recognition. 

Deciphering the sequence variability in TIMPs from evolutionary distant organisms could support the development of engineered TIMPs with unique features in terms of binding affinity and patterns of selectivity.

Therefore, TIMP domains could be selectively optimized by integrating amino-acid variations, which provide additional features or specific patterns of inhibition, as obtained in “ancient TIMPs”, with structural constraints operating in scaffolding, in which the N and C-terminal domains could cooperate to achieve the desired binding characteristics.

## Figures and Tables

**Table 1:** TIMPs from different metazoans possessing unusual helices in the N-domain and exposure propensity in terms of RSA.

	Species		AA Residues in Helix	Accession
Primate	*Pan troglodytes*	TIMP-4	FEKV **EBEE**	XP_516284.1
		TIMP-3	KMPKV **IEIBB**	XP_515097.2
Rodent	*Rattus norvegicus*	TIMP-1	FDA **EEI**	NP_446271.1
		TIMP-4	FEKAK **IBEEE**	NP_001102863.1
Bird	*Columba livia*	TIMP-4	FEKL **IBEE**	EMC77392.1
Turtle	*Chelonia mydas*	TIMP-4	FEKV **EBEE**	XP_007056544.1
Sea urchin	*Strongylocentrotus purpuratus*	TIMP-3	EKLKH **EEBEE**	XP_781027.1
Insect	*Stegodyphus. mimosarum*	TIMP	EKARRA **EEEBEE**	KFM62985.1
Molluscs	*Crassostrea gigas*	TIMP-1	SLLGS **EEBIE**	AAT73610.1
		TIMP-2	KGSSLL **IBBEEI**	NP_001292265.1
	*Tegillarca granosa*	TIMP	PAFEEL **EEEBEE**	AFB81539.1
Cnidarian	*Hydra magnipapillata*	TIMP3a	NPSYRFNLQQIH **EIIIEEEIEEBB**	449680372
		TIMP3b	YQFNL **EEIIEE**	221128951
		TIMPa	NLQQIH **EBEEBI**	449683625

TIMPs and corresponding RSAs are according to [10]. Amino-acid residues structuring the helices are shown, while the corresponding RSA behaviour is shown in bold: exposed residue (E), intermediate residue (I) and buried residue (B).

## Data Availability

Not applicable.
